# Scan, Screen, Support—A Digital Pathway for Assessing Supportive Care Needs in Oncology: Implementation Study

**DOI:** 10.2196/82392

**Published:** 2026-03-19

**Authors:** Theres Fey, Nicole Thurner, Ulrike Haidn, Friederike Mumm, Birgit Haberland, Rachel Wuerstlein, Sebastian Theurich, Georg Wolfrum, Marie-Louise Troidl, Susan Müller, Claudia Bausewein, Timo Schinköthe, Volker Heinemann, Nicole Erickson

**Affiliations:** 1 Comprehensive Cancer Center (CCC Munich LMU), LMU Hospital, Pettenkoferstr. 8a, München, Bavaria, 80336, Germany, 49 89440075218; 2 Bavarian Cancer Research Center (BZKF), Germany; 3 Department of Medicine III, LMU Hospital, München, Bavaria, Germany; 4 Department of Palliative Medicine, LMU Hospital, München, Germany; 5 Department of Obstetrics and Gynecology, LMU Hospital, München, Germany; 6 Department for Medical Technology and IT, LMU Hospital, München, Germany; 7 CANKADO GmbH, München, Germany; 8 Research Center Smart Digital Health, University of the Bundeswehr Munich, München, Germany

**Keywords:** electronic patient-reported outcomes, ePROs, patient-reported outcomes, PROs, digitalization, routine screening, digital screening, supportive cancer care, psycho-oncology, distress, nutrition screening, palliative care

## Abstract

**Background:**

Electronic patient-reported outcome tools have the potential to enhance supportive care in oncology and support the timely and accurate identification of patients’ needs.

**Objective:**

This study aimed to develop, implement, and evaluate a user-friendly, web-based digital screening tool at a German Comprehensive Cancer Center that systematically and efficiently assesses the supportive care needs of patients with cancer and enables direct referral to appropriate supportive services through seamless integration with the hospital information system.

**Methods:**

An interdisciplinary team collaborated with the IT department and the company CANKADO, an electronic patient-reported outcome provider, to create a 14-item digital questionnaire. The tool incorporated validated instruments, such as the Distress Thermometer, the Nutrition Risk Screening, and a short form of the Integrated Palliative Care Outcome Scale, aligned with German Cancer Society certification criteria. Patients accessed the questionnaire via QR codes. Screening results were automatically transferred to the hospital information system, where supportive care requests (SCRs) were generated automatically if indicated.

**Results:**

Between June 2024 and May 2025, a total of 8855 QR codes were generated. Of these, 4909 questionnaires were complete and valid for analysis. This information produced 3324 SCRs. Digital screenings resulted in an SCR in 22.4% of cases for psycho-oncology, 18.7% for nutrition, and 27.6% for palliative care. The digital screening maintained or slightly improved screening rates compared to prior methods.

**Conclusions:**

The implementation of a digital supportive care screening was feasible and effective within the Comprehensive Cancer Center setting. Future efforts should focus on overcoming barriers for patients with limited digital access or capabilities to ensure the delivery of equitable supportive care.

## Introduction

Contemporary oncology acknowledges that supporting patients with cancer through their journey is essential. The diagnosis and treatment of cancer entail physical challenges as well as psychological and social burdens. Consequently, providing care that holistically addresses these multifaceted needs is of precedence [1,2]. Ensuring that individualized, need-based care is integrated into the treatment process requires systematic assessment of these needs [3]. Patient-reported outcome (PRO) assessments in the context of psycho-oncological and palliative care screenings are already established in Comprehensive Cancer Centers (CCCs) across Germany and are incorporated into clinical guidelines [4-8].

The German Cancer Society recommends the implementation of an interdisciplinary screening procedure to evaluate counseling needs as an integral component of basic oncological assessment [9]. Historically, supportive needs were assessed via interviews or paper-based questionnaires. These methods, however, face limitations with regard to real-time data collection, structured documentation, and accessibility [10]. Thus, digital solutions for the collection of PROs have the potential to optimize support for patients with cancer by facilitating fast and precise identification of support needs while reducing the burden on health care professionals (HCPs) [11-13]. Furthermore, electronic patient-reported outcomes (ePROs) interventions have been shown to have a positive impact on health-related quality of life and symptom burden [13,14].

Notably, digital tools enable continuous and standardized assessment of patient needs across various treatment stages. This consistency is crucial, given that a patient’s need for supportive care often evolves throughout the disease trajectory [11].

We therefore aimed to develop and implement a user-friendly, web-based digital screening tool to enable rapid and accurate collection of patient needs within a German CCC. The tool was designed to facilitate direct access to supportive services while ensuring seamless integration with the hospital information system (HIS). In parallel, challenges related to the digital implementation within routine clinical practice were examined.

## Methods

### Implementation Design

The implementation-oriented design of the project was guided by the EPIS (Exploration, Preparation, Implementation, Sustainment) framework, which provides a structured approach to implementing innovations in health care settings [15,16]. The project comprised 4 key phases corresponding to the EPIS framework.

#### Exploration

During the exploration phase (2017‐2020), various platforms for collecting PROs were evaluated within the context of clinical studies at the CCC [17,18], as well as in the Department of Gynecology [19,20] and the Department of Hematology and Oncology [21]. Following this evaluation phase, the CANKADO platform was selected as a digital platform for web-based questionnaire delivery due to its ease of use, sustainability, and affordability [22]. An interdisciplinary working group was established, comprising experts from the fields of medicine and oncology, nutrition science, psycho-oncology, palliative care, and IT. Initial preparations for the implementation of the digital screening system began in 2020 but were delayed as a result of the COVID-19 pandemic. Compliance with information security and data protection regulations was ensured at all times (Textbox 1).

Textbox 1. **Implementation steps using the Exploration, Preparation, Implementation, Sustainment (EPIS) framework.**

**Exploration (2017-2020)**

- Formation of an interdisciplinary working group
- Development of a requirements catalog for a browser-based questionnaire platform
- Vendor screening and market research
- Pilot projects with 2 vendors
- Decision to partner with CANKADO’s ePRO system
- Feasibility studies with CANKADO

**Preparation (2020-2023)**

- Questionnaire development
- Establishment of required IT infrastructure (automated patient registration, integration into the HIS, generation of QR codes)
- Programming and technical implementation within CANKADO and the HIS
- Data protection measures

**Implementation (2023-2025)**

- Pilot implementation
- Adaptation of the questionnaire based on pilot results
- Engagement and endorsement by medical and nursing leadership
- Training of HCPs in oncological departments
- Hospital-wide rollout
- Development of patient information materials

**Sustainment (since 2025; ongoing)**

- Refresher trainings for HCPs
- Continuous monitoring of screening uptake and effectiveness
- Implementation of support measures for patients with barriers to digital participation
- Evaluation of acceptance and use by HCPs (not part of this manuscript)
- Evaluation of patient acceptance (not part of this manuscript)

#### Preparation

The preparation phase involved the development of the PRO questionnaire, digital implementation, and ensuring data protection compliance.

##### Questionnaire Development

A tailored questionnaire, composed of existing validated short-form questionnaires, was developed in close coordination with the relevant disciplines (Department of Psycho-Oncology, Nutrition, and Palliative Care). This method ensured alignment with the certification requirements of the German Cancer Society (see Table 1). The National Comprehensive Cancer Network Distress Thermometer, the short version of the Integrated Palliative Care Outcome Scale (IPOS), and the Nutrition Risk Screening (NRS) 2002 served as the primary sources for item selection [23-26].

**Table 1.** Questionnaire.

Category and validated questionnaire		Question
Psycho-oncology (2 questions)^a^		
	NCCN^b^ Distress Thermometer	1. Please circle the number (0‐10) that best describes how much distress you have been experiencing in the past week including today.
	Request	2. Would you like psychological counseling? Yes / No / No answer
Nutrition (5 questions)^c^		
	NRS^d^ and PG-SGA^e^ SF	3. I currently weigh about ______ kg.
	NRS and PG-SGA SF	4. I am about ______ cm tall.
	NRS and PG-SGA SF	5. Three months ago, I weighed about ______ kg.
	NRS and PG SGA SF	6. How much did you eat last week compared to your usual amount? (Please mark a value between 0%‐150%)
	Request	7. Would you like nutritional counseling? Yes / No / No answer
Palliative care (6 questions)^f^		
	IPOS^g^ (introductory text)	For each symptom, please tick the box that best describes how it has affected you over the past 3 days.
	IPOS	8. Pain: Not at all (0), Slightly (1), Moderately (2), Severely (3), Overwhelmingly (4)
	IPOS	9. Shortness of breath: Not at all (0), Slightly (1), Moderately (2), Severely (3), Overwhelmingly (4)
	IPOS	10. Poor mobility: Not at all (0), Slightly (1), Moderately (2), Severely (3), Overwhelmingly (4)
	IPOS	11. Have you been feeling anxious or worried about your illness or treatment? Not at all (0), Occasionally (1), Sometimes (2), Most of the time (3), Always (4)
	IPOS	12. Have any of your family or friends been anxious or worried about you? Not at all (0), Occasionally (1), Sometimes (2), Most of the time (3), Always (4)
	IPOS	13. Have any practical problems resulting from your illness been addressed? (such as financial or personal) Problems addressed/no problems (0), Problems mostly addressed (1), Problems partly addressed (2), Problems hardly addressed (3), Problems not addressed (4)
General (1 question)^h^		
	IPOS	14. How did you complete this questionnaire? On my own / With help from a friend or relative / With help from a member of staff

a A supportive care request (SCR) is automatically generated if the Distress score is >4 and psychological counseling is requested or not specified, or if psychological counseling is requested regardless of the Distress score.

b NCCN: National Comprehensive Cancer Network.

c An SCR is automatically generated if weight loss is >5% within 3 months, or BMI <20.5 kg/m², or food intake <75%, and nutritional counseling is requested or not specified, or if nutritional counseling is requested regardless of nutritional values.

d NRS: Nutrition Risk Screening.

e PG-SGA: Patient-Generated Subjective Global Assessment.

f An SCR is automatically generated if the IPOS-5 score is ≥8 or the IPOS-6 score is ≥10.

g IPOS: Integrated Palliative Care Outcome Scale.

h This question was taken from the IPOS but applies to the entire questionnaire.

For the identification of malnutrition and the need for nutrition interventions, the NRS was already the established screening tool in the hospital. However, the questions in the NRS were designed and validated to be completed by HCPs. For example, the first question asked whether the body mass index is less than 20.5 kg/m^2^, which cannot be answered by a patient using a PRO tool. Therefore, the risk assessment points from the NRS tool were reworded analog to the wording validated in the Patient-Generated Subjective Global Assessment-Short Form, which is a validated PRO tool [23]. The cut-off points for the risk assessment remained in line with the NRS. Based on recent research results, the questions of the IPOS were adapted from IPOS-5 to IPOS-6 in April 2025 [27,28].

Key requirements for the overall questionnaire included brevity, ease of understanding for patients, and applicability across various stages of the disease. The final questionnaire, consisting of 14 items, serves as a prescreening tool. If necessary, a more detailed screening can be conducted by the relevant medical specialty. Regardless of the total score, patients also have the opportunity to actively express a desire for counseling services in the area of psycho-oncology or nutrition (items 2 and 7 of the questionnaire). This question was not included for palliative care.

A screening was considered valid if 1 or more of the following criteria were met: for the distress screening, at least 1 question had to be answered; for the nutrition screening, at least 1 question had to be answered; for the palliative screening using IPOS-5, at least 2 questions had to be answered; and for the palliative screening using IPOS-6, at least 3 questions had to be answered. Screenings that were accessed but had no items completed were not included.

##### Digital Implementation

The CANKADO ePRO system was initially developed for collecting PRO in clinical trials [20,29-31]. It utilizes a cloud-based electronic health record with a role-based access concept. Development and operation are carried out under ISO 27001 certification. Patients can access their ePRO questionnaires through personalized access via the web or an app. In addition, specific questionnaires can be accessed directly via a QR code. These QR codes are implemented to make access possible regardless of the device and without an additional app. Alternatively, the questionnaire can be completed via a tablet provided by the center and secured by kiosk software.

Based on this existing architecture, the CANKADO ePRO-based questionnaire was integrated into the HIS for use in routine clinical operations in oncology. The aim was to automate the entire process for HCPs and make it fully mappable within the HIS. To enable the integration of CANKADO ePRO into the hospital’s existing SAP-based HIS, several components were required: (1) retrieval of a QR code linking to the digital screening questionnaire, (2) receipt of the completed digital screening as both a PDF document and structured data, (3) display of an overview of the most recent survey within a subsystem, and (4) storage of data for subsequent evaluation.

Within the existing HIS, a button was integrated to generate and retrieve the QR code. This request is routed via a Python-based HTTPS service to CANKADO. When a digital screening questionnaire is triggered in the HIS for a specific patient (based on the admission number) for the first time, this initiates the creation of a new, pseudonymized patient file in CANKADO. This file is assigned a generated identifier, which is then sent back to the HIS. At the same time, the corresponding questionnaire is assigned to the newly created patient number and coupled with a QR code, which is transmitted to the HIS. A base64-encoded image of the QR code is securely retrieved and displayed through the HTTPS service of the hospital. The generated QR code can be printed by the nursing staff and then handed to the patient, who completes the questionnaire digitally. The QR code can be regenerated for further assessments of the same patient.

After completion of the questionnaire, the data are automatically transmitted to the HIS. For security reasons, the data are received by a server located in a demilitarized zone (DMZ). The assignment to the real person takes place exclusively within the HIS. For each patient, the PDF document of the completed digital questionnaire is archived with a timestamp in the HIS. The data derived from the digital screening are additionally stored in accordance with the requirements set by the auditing bodies of the certified cancer centers in Germany. An entry in the documentation system is visible to show that a digital screening was performed. Based on the answers, a supportive care request (SCR) is automatically generated via web services (as shown in Table 1). In the final step, a physician reviews the clinical indication and grants approval.

HCPs can additionally be granted permission to access all deidentified patient records via the CANKADO web portal. There, they can view the completed questionnaires and calculated scores and export them if necessary. The structured data from the HIS are provided in JSON format and stored in a MongoDB database to facilitate efficient data evaluation and analysis. The following data can thus be analyzed in a structured manner: number of screenings performed and number of SCRs by the special departments (psycho-oncology, nutrition, palliative care).

##### Data Protection

The entire process was conducted in accordance with applicable data protection regulations. Approval was granted by the institutional data protection officer prior to implementation. A detailed data processing overview is shown in Table 2.

**Table 2.** Data processing overview.

Steps	Collection of personal data	Who accesses the data?	Duration of storage
Initiation			
ePRO creation by CANKADO	No personal data collected	No access	No storage
Creation of user accounts for medical staff	Data collected: Email address Name (optional) First name (optional) Place of employment Address of workplace Employment relationship (eg, study nurse)	Access by: Trained CANKADO staff	Until the project ends, unless the account is no longer used or the user leaves the organization. Otherwise, until the user deletes the account.
Adding a patient	Data collected: Patient ID (automatically generated by the system) Year of birth (for studies) Other basic data (optional)	Access by: Trained CANKADO staff (only Patient ID) Medical staff from the clinic/practice	Until the project ends, unless legal retention periods must be observed. Archiving and storage until the end of legal periods.
Documentation of patient data	Data collected: Project-dependent health data (eg, health status)	Access by: Medical staff from the clinic/practice	Until the project ends, unless legal retention periods must be observed. Archiving and storage until the end of legal periods.
Project-specific evaluation of patient data	Data collected: Purpose-related analysis of anonymized patient data	Access by: Selected staff from the clinic/practice	Until the project ends, unless legal retention periods must be observed. Archiving and storage until the end of legal periods.
End			

### Implementation

**Figure 1.**
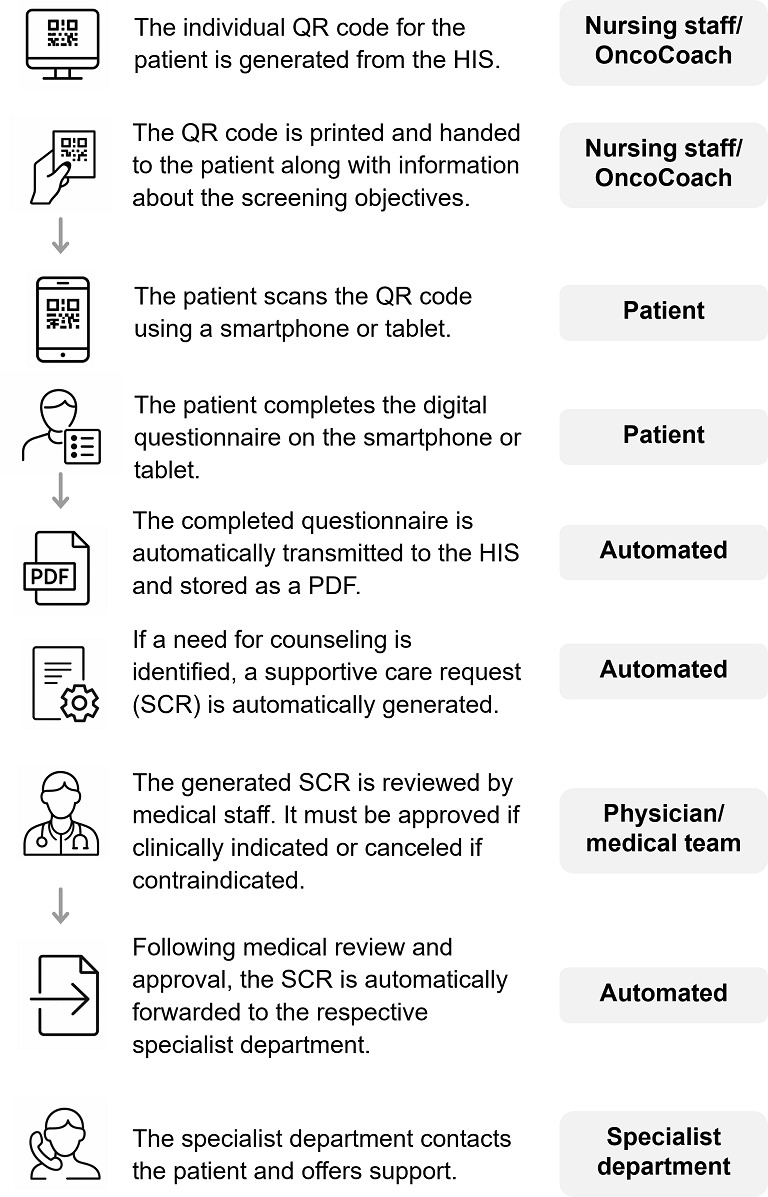
Screening procedure. HIS: hospital information system.

The pilot phase was conducted from April to May 2023 on an outpatient oncology ward. Insights gained during this period regarding the process flow, the stakeholders involved, and the questionnaire itself were used to refine the instrument and address procedural challenges. Starting in June 2024, the interdisciplinary digital screening was rolled out across all oncology inpatient wards and several outpatient units, marking its integration into routine clinical care. To ensure consistent implementation, training sessions were organized with nursing staff and physicians in each department beginning in May 2024. These sessions were systematically conducted by designated, qualified CCC employees. The screening process is shown in Figure 1. The process, along with the designated responsibilities, was outlined in a standard operating procedure made accessible to all HCPs. Barriers and challenges were regularly addressed and evaluated during recurring meetings of the working group, where ongoing developments were also critically discussed.

The implementation of digital screening was first piloted in a single outpatient clinic. This clinic was selected due to its relatively new organizational structure, where routine, comprehensive distress screening had not yet been fully implemented at the start of the project. Distress screening rates were compared between two equivalent six-month periods: July to December 2023 (analog screening only) and July to December 2024 (a combination of analog and digital screening).

### Sustainment

The sustainment phase focused on embedding the digital screening into routine clinical practice and ensuring long-term adoption. HCPs’ acceptance was assessed through an evaluation in early 2025, and ongoing support structures—including patient navigators for patients facing digital barriers—were introduced to maintain equity and inclusivity. Structured data storage and integration with hospital systems allow for continuous monitoring of screening uptake and effectiveness.

### Ethical Considerations

This study used fully anonymized data analyses and involved no intervention. All data were extracted from the hospital patient information system and fully anonymized prior to analysis, precluding any direct or indirect patient reidentification. Thus, the local ethical committee provided written confirmation that no formal ethical consultation was necessary (application number 24‐0535 KB). An informed consent was not required, and no compensation was provided to participants.

## Results

### HCP Training

Between May 2024 and May 2025, a total of 48 training sessions were conducted at 32 oncology wards and departments within the LMU Hospital. Thirty-two training sessions were conducted for nursing staff, and 13 were conducted for medical staff. Additionally, 3 training sessions were held for specialized departments involved in providing supplementary counseling services (psycho-oncology, nutrition, and the palliative care team). Overall, 43 of 59 (72.9%) organizational units involved in the care of patients with oncology received training on digital screening procedures. In this phase, the focus was on inpatient units and day clinics; most outpatient areas were not yet included.

### 12-Month Implementation Period

After the implementation of the digital screening, a total of 8855 QR codes (738 on average per month) were generated between June 2024 and May 2025. A total of 4909 (55.4%) questionnaires (409 on average per month) were completed and deemed valid. This means that, on average, 329 (44.6%) QR codes per month were either not accessed or accessed without any items being answered. In 946 (10.7%) instances, the questionnaire was accessed via the QR code, but no items were filled out. A total of 3822 (77.9%, 3822/4909) evaluable screenings were performed by the patient only once, while 1087 of 4909 (22.1%) evaluable screenings were performed at least twice.

**Figure 2.**
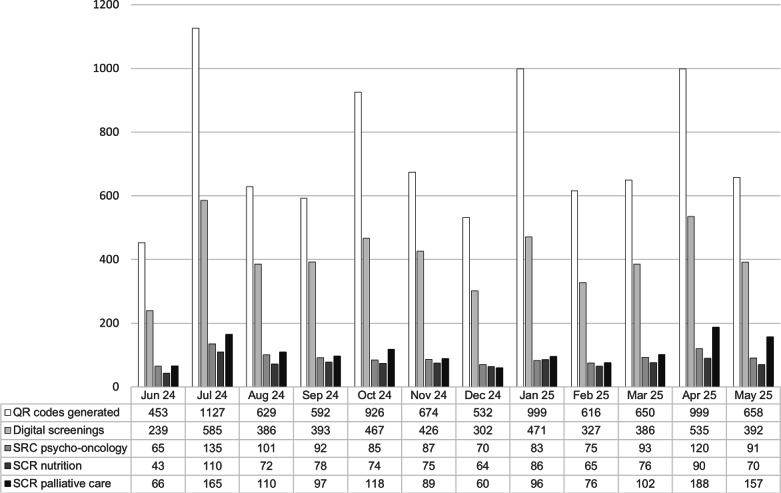
Development of the generated QR codes, the screenings conducted (at least 1 question answered; screenings that were accessed but had no items completed were not included), and the supportive care requests (SCRs) created in the period from June 2024 to May 2025.

Among the 4784 responses to the question about how the questionnaire was completed, 80.1% (n=3831) were completed independently by the patients, 15.6% (n=746) with assistance from relatives, and 4.3% (n=207) with support from staff. The screenings resulted in a total of 3324 SCRs, including 1097 (22.4%) for psycho-oncology, 903 (18.7%) for nutritional counseling, and 1324 (27.6%) for palliative care. The analysis per month is shown in Figure 2. The number of QR codes created was highest at the beginning of each quarter.

**Figure 3.**
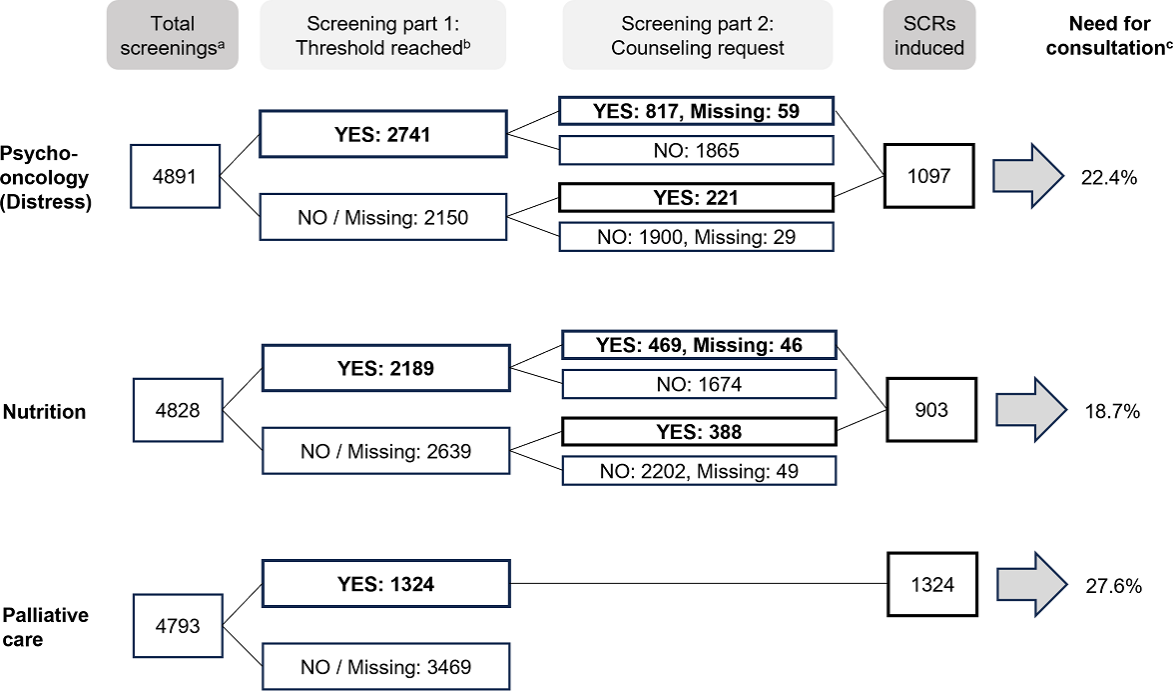
Evaluation of questionnaires in the areas of psycho-oncology (distress screening), nutrition screening, and palliative care screening, as well as the initiated SCRs (supportive care requests). ^a^A screening was considered valid when 1 or more of the following fields were completed: distress screening, at least 1 question answered; nutrition screening, at least 1 question answered; palliative screening (IPOS-5), at least 2 questions answered; palliative screening (IPOS-6), at least 3 questions answered. ^b^Screening thresholds are detailed in Table 1. ^c^The need for consultation is determined by the number of initiated SCRs in relation to the total number of screenings performed.

In total, 4891 patients’ screenings indicated a distress value and/or the patient requested a psychosocial consultation. Of these, 56% (n=2741) had an increased distress value of more than 4 points (see Figure 3). An SCR was triggered for 32% (n=876) of these screenings, which had an increased distress score. Accordingly, no SCR was triggered for 68% (n=1865) of the people with an increased distress score, as they had rejected psycho-oncology counseling. Fifty-nine SCRs were induced due to an increased distress value, and there was no information available on whether the patient had a desire for counseling. The majority of SCRs (n=817, 74.5%) were induced by a combination of an increased distress value and a request for counseling by the patient on the digital screening. Less frequently, the SCR was triggered solely due to the request for counseling (n=221, 20.1%), as the distress value was below the threshold rate and/or not specified. The overall demand for psycho-oncological counseling was 1097 out of 4891 (22.4%).

A total of 4828 questionnaires were completed with at least 1 item of the nutrition screening and/or the patients provided information about their need for consultation. Of these, 45.3% (n=2189) met the nutrition-specific thresholds. An SCR was triggered for 23.5% (n=515) of these screenings. Accordingly, no SCR was triggered for 76.5% (n=1674) of the screenings with corresponding nutritional values, as the patient had rejected nutrition counseling. A total of 469 of 903 (51.9%) of the SCRs for nutrition were triggered because the threshold value was exceeded and the patient requested nutrition counseling. Additionally, 388 of 903 (43%) SCRs were patient requests for nutrition counseling although the threshold value was not reached or specified. Furthermore, 46 of 903 (5.1%) SCRs were induced due to an increased nutrition threshold value, while there was no information available on whether the patient had a desire for counseling. In summary, the overall need for nutritional counseling was 903 of 4828 (18.7%).

A total of 1324 of 4793 (27.6%) of the patients who completed or partially completed the screening for palliative care (n=4793) also had an elevated IPOS score.

Overall, 1 or more counseling needs were identified and triggered in 3072 of 4909 (62.6%) of the screenings. Accordingly, 1837 of 4909 (37.4%) of the screenings revealed no unmet needs.

### Comparison of Development: Analog vs Digital Derived From a Single Outpatient Clinic Within the LMU Hospital

**Figure 4.**
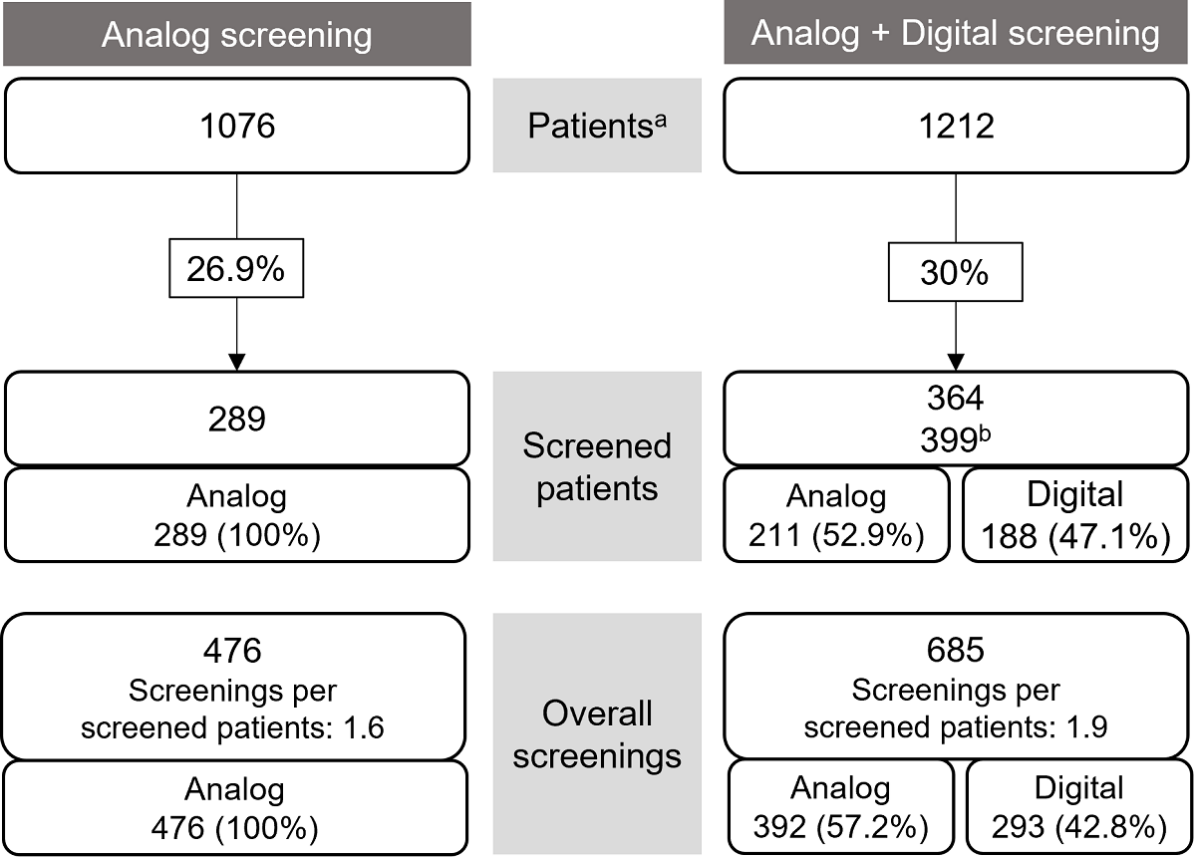
Comparison of distress screenings conducted over two 6-month periods at the oncological outpatient clinic. From July to December 2023 (6 months), distress screening was still performed using paper forms (analog screening, left side). The analysis on the right includes data collected immediately after the implementation of the digital screening, during a transitional phase in which both paper-based and digital methods were used (digital + analog screening, July-December 2024). ^a^Number of patients undergoing treatment in the respective period. ^b^Some patients were screened through both analog and digital methods during the specified period and therefore were counted twice.

Distress screening rates were compared in 2 comparable 6-month periods: between July and December 2023 (analog screening only) and July to December 2024 (a combination of analog and digital screening). All relevant staff of the outpatient clinic were trained in the use of digital screening in May 2024. Since the transition to digital screening was gradual, analog screenings continued to be conducted alongside the digital ones during this period. Nevertheless, after the introduction of digital screening, the percentage of screened patients increased from 26.9% to 30%. In the 6 months following the implementation, 57.2% of the distress screenings were still conducted using the analog method, while 42.8% were carried out using the digital screening (see Figure 4). The number of screenings per screened patient increased from an average of 1.6 to 1.9 after the implementation.

## Discussion

### Principal Findings

The study demonstrates the successful implementation of a structured, standardized, and ePRO-driven digital screening process for assessing supportive care needs in oncology at LMU University Hospital. The screening tool enables the systematic collection of data in accordance with the screening requirements set by the German Cancer Society (DKG). It is fully integrated into routine clinical workflows via the HIS. A central design principle was the use of a concise questionnaire limited to 14 items, ensuring feasibility and acceptability in everyday clinical practice. Shorter questionnaires have been associated with higher participation rates, reduced respondent burden, and improved inclusivity across diverse patient populations [32-34]. A comparable implementation approach has been described by Maurer et al [35], who similarly integrated ePRO-based supportive care screening into routine clinical workflows, albeit using a more extensive questionnaire. Although the screening pathway was developed within the framework of German Cancer Society certification, the underlying concept is not restricted to the German health care setting. In fact, the concept can be adapted to other national contexts with comparable digital infrastructures and care pathways. Similar ePRO-driven screening strategies have been reported from other countries, demonstrating the feasibility of embedding digital needs assessments into routine cancer care in diverse health system contexts [36,37].

### Feasibility, Adherence, and Screening Outcomes

The questionnaire incorporates short-form PRO versions of the Distress Thermometer, the NRS with wording from the Patient-Generated Subjective Global Assessment, and the IPOS. As a prescreening tool, it supports targeted follow-up assessments by the respective specialties. During the first 12 months, 8855 QR codes were generated, and 4909 (55.4%) screenings were completed. According to the literature, the proportion of patients completing standardized distress or supportive care needs screenings typically ranges from 50% to 70%, depending on the clinical setting and the methodology used [38-40]. The adherence observed in our study is therefore comparable to these published rates. However, the true adherence rate may be underestimated, as some QR codes may not have been distributed for various reasons, such as patient absence. In addition, a number of QR codes were generated for training purposes or as part of system testing during the implementation phase.

Across all completed screenings, the identification of unmet needs resulted in SCRs in 22.4% of screenings for psycho-oncology, 18.7% for nutrition, and 27.6% for palliative care. These findings are broadly consistent with prior studies reporting substantial unmet needs among patients with oncology, although direct comparisons are limited by differences in screening scope and methodology [41,42]. Previous research reported that 23% of patients with cancer were newly referred to palliative care [43]. Our study observed a higher level of need, which can be attributed to the requirement for physician-led final approval of referrals. This finding also reflects the substantial symptom burden in a patient cohort that was not preselected based on medical status.

### Equity, Digital Access, and Patient Support

Despite the advantages of digital screening, patient-related barriers may limit participation. Older age, poor health status, lack of access to digital devices, and limited digital literacy can reduce adherence and equity. To mitigate these barriers, paper-based screenings continue to be offered for selected patients. In addition, targeted measures have been introduced to improve digital inclusivity. Additional informational material has been created to explain the purpose and process of the screening, helping patients understand its importance and encouraging participation. Pilot projects are currently underway in several organizational units, where patients facing barriers to digital participation receive active support from trained patient navigators. These navigators play a central role in enabling patients—regardless of age, health status, or digital literacy—to benefit from the digital screening pathway. Institution-provided tablets are used as supportive tools to facilitate access and completion of screenings. Hybrid models combining digital tools with human support have been shown to enhance participation and effectiveness, highlighting the value of patient navigators [44].

### Impact on Screening Rates in Routine Care

Screening rates were evaluated on a single outpatient ward before and after the implementation of digital screening. Following implementation, screening rates were maintained or slightly increased (26.9% vs 30%), largely through a combined use of digital and paper-based methods. Maintaining hybrid workflows ensured continuity of care and inclusivity during the transition phase.

Reported screening rates reflect all patients treated during the respective periods and are therefore lower than DKG benchmarks for newly diagnosed primary patients [45,46]. In 2024, the LMU Oncology Center achieved the required 65% threshold for distress screening, to which the digital implementation may have contributed. Distress assessments documented outside standardized reporting structures could not be captured in the analysis.

### Facilitators and Barriers to Implementation

Several facilitators supported successful implementation. The DKG requirement for structured screening provided a strong regulatory framework. In addition, implementation was supported by hospital leadership, the IT department, and the technology provider. The allocation of dedicated personnel enabled systematic training, coordination, and continuous refinement of the system. Digitalization also yielded operational benefits. A unified questionnaire simplified workflow, automated data transfer, eliminated manual data entry and document archiving, and ensured the immediate availability of screening results in the HIS. The automated generation of SCRs based on predefined thresholds further reduced administrative burden and improved transparency.

Nevertheless, several barriers were encountered. Generating QR codes initially appeared more time-consuming than distributing paper questionnaires, and the downstream time savings were not always immediately apparent to staff. Comprehensive and repeated training sessions were, therefore, essential, consistent with prior implementation research [47-49]. Moreover, purely digital approaches cannot fully reach all patient groups. Approximately 20% of patients required assistance to complete the questionnaire, highlighting the need for supportive strategies.

### Comparison With International Implementations

International experience with digital distress and supportive care screening reveals similar challenges. In the United States, electronic screening is often integrated into patient portals or electronic health records; however, documentation gaps and limited follow-up remain common [36]. In the United Kingdom, digital Holistic Needs Assessments are available and easy to use, but their application in everyday clinical practice is limited. The main problems include a lack of commitment, time constraints within the care team, and insufficient feedback of ePRO results into concrete care decisions, which reduce motivation to use them among both patients and professionals [50]. Australia has technically mature, digitally integrated screening workflows with high initial participation rates. Nevertheless, there are issues with acceptance in subsequent courses, particularly with repeated screenings, as well as uncertainties among staff regarding how to handle high distress values and their implications. Here, too, usage depends heavily on training, local leadership, and clear responsibilities [37,51].

In Asia, digital ePRO approaches vary considerably. In Japan, distress screening is formally established but is often only partially digitized and has limited structured follow-up [52]. In South Korea and China, there are significant barriers to acceptance: low routine use, cultural reluctance to seek psychosocial help, and low acceptance rates after a positive ePRO screening [53,54]. Singapore is an exception, as digital distress ePROs are closely linked to a multidisciplinary care model and thus achieve a high level of acceptance [55]. In Europe, the Netherlands exemplifies successful implementation when digital screening is mandatory, systematically discussed, and directly linked to care pathways [56]. Overall, international evidence suggests that technical availability alone is insufficient; sustained use depends on clear workflows, automated follow-up, leadership support, and perceived clinical relevance [57].

### Generalizability and Transferability

Although conducted at a single CCC, several features support transferability. The screening content is based on internationally validated instruments and guideline-aligned supportive care domains. Technically, the CANKADO platform is HIS-agnostic. Interfaces are implemented via standardized web-based application programming interfaces and enable integration with heterogeneous hospital IT environments beyond the SAP-based infrastructure used at LMU Hospital. Structured data exchange enables flexible mapping to local documentation and reporting requirements.

The system complies with the General Data Protection Regulation and the more stringent French Hébergeurs de Données de Santé framework, supporting international applicability. While the depth of integration achieved at LMU Hospital has not yet been replicated elsewhere, the platform is already used in multiple international settings [19,30,58,59]. Future multicenter studies are needed to evaluate scalability, resource requirements, and contextual adaptations across different health care systems.

### Limitations

Despite the demonstrated feasibility and advantages of digital supportive care screening in oncology, several limitations should be acknowledged. Technical challenges, such as temporary connectivity issues, system maintenance, or software-related disruptions, may intermittently affect access to the digital platform and result in delayed or incomplete data capture. In addition, the implementation and maintenance of a secure, interoperable digital screening infrastructure require substantial technical, administrative, and human resources. Ensuring ongoing compliance with data protection regulations and IT security standards represents a continuous organizational effort.

A key limitation relates to equity and accessibility. Digital screening inherently excludes certain patient groups, particularly individuals without access to suitable digital devices or with limited digital literacy. Older patients, those with cognitive or physical impairments, and patients with advanced disease or severe symptom burdens may face additional barriers to independent participation. Although supportive measures, such as paper-based alternatives and assisted completion, were available, these barriers may have reduced the inclusivity and representativeness of the screening results.

Human resource constraints also represent a relevant limitation. High workload among HCPs, especially in nursing and medical staff, may delay the initiation of screening or the review and approval of triggered SCRs. In particular, delays in physician review of SCRs may hinder timely referral to counseling services and continuity of care. At present, systematic evaluation of approved SCRs, completed counseling sessions, or canceled referrals is not yet possible within the existing data structure; however, this functionality is currently under development and will be addressed in future analyses.

Furthermore, the digital questionnaire currently does not adequately address linguistic and cultural diversity. The lack of multilingual versions may limit accessibility for patients with limited proficiency in the primary language of care and may reduce the effectiveness of the screening in culturally diverse populations. While digital implementation allows relatively straightforward adaptation to additional languages, this feature has not yet been fully implemented at the time of evaluation.

Finally, the study was conducted at a single academic CCC with a high degree of digital maturity and IT integration. Although the technical architecture and conceptual approach are transferable, implementation depth, available resources, and organizational workflows may differ substantially across institutions. These contextual factors should be considered when interpreting the findings and underscore the need for future multicenter evaluations.

### Implications for Clinical Practice

The findings of this study provide several practical implications for the implementation of digital supportive care screening in routine oncology practice. First, successful implementation requires careful preparation, clear governance structures, and the allocation of sufficient technical and human resources. Defining the scope of supportive care domains and selecting a concise questionnaire that balances clinical relevance with feasibility are essential to facilitate integration into daily workflows.

Comprehensive and role-specific training programs for HCPs are critical. Initial training should be complemented by regular refresher sessions to maintain engagement, address uncertainties, and integrate system updates. Assigning clear responsibilities to specific professional groups—for example, nursing staff for screening initiation and physicians for SCR approval—supports accountability and process reliability. Introducing the screening initially in motivated units or pilot wards may foster early adoption and generate positive momentum for broader rollout.

Maintaining flexibility in screening modalities is particularly important during implementation phases. Hybrid workflows that combine digital and paper-based screening can ensure continuity of care and inclusivity, especially for patients facing access barriers. From a technical perspective, screening data should be available both as archivable PDF documents and as structured data within the HIS to support documentation, quality assurance, and secondary analyses. Systems should also be adaptable to future modifications of questionnaire content or screening frequency.

Equally important is the proactive consideration of equity and accessibility. Implementation strategies should explicitly address patients with limited digital skills, physical impairments, or language barriers. Support structures such as patient navigators, assisted completion, and the provision of institution-owned devices can substantially enhance digital participation. Finally, digital screening will only be sustainably adopted if both patients and HCPs perceive clear clinical benefits. Automated follow-up processes, timely referrals, and visible improvements in patient care are, therefore, essential to reinforce acceptance and long-term use.

### Conclusion

A digital oncological screening in the supportive care setting was successfully implemented at a German CCC. Automated integration into the HIS enabled efficient identification of supportive care needs, reduced administrative burden, and supported patient-centered care. Continuous training and standardized procedures were critical to success. Future initiatives will focus on improving digital equity and accessibility to ensure that all patient groups benefit from supportive oncology care.

## Abbreviations

CCC: Comprehensive Cancer Center
EPIS: Exploration, Preparation, Implementation, Sustainment
ePRO: electronic patient-reported outcome
HCP: health care professional
HIS: hospital information system
IPOS: Integrated Palliative Care Outcome Scale
NRS: Nutrition Risk Screening
PRO: patient-reported outcome
SCR: supportive care request
